# Pathological Study of Facial Eczema (Pithomycotoxicosis) in Sheep

**DOI:** 10.3390/ani11041070

**Published:** 2021-04-09

**Authors:** Miguel Fernández, Valentín Pérez, Miguel Fuertes, Julio Benavides, José Espinosa, Juan Menéndez, Ana L. García-Pérez, M. Carmen Ferreras

**Affiliations:** 1 Departamento de Sanidad Animal, Facultad de Veterinaria, Universidad de León, C/Prof. Pedro Cármenes s/n, E-24071 León, Spain; vperp@unileon.es (V.P.); jespic@unileon.es (J.E.); mcfere@unileon.es (M.C.F.); 2 Instituto de Ganadería de Montaña (IGM), CSIC-Universidad de León, Finca Marzanas s/n, E-24346 León, Spain; julio.benavides@csic.es; 3 Department of Animal Health, NEIKER-Basque Institute for Agricultural Research and Development, Basque Research and Technology Alliance (BRTA), Parque Científico y Tecnológico de Bizkaia, P812, E-48160 Derio, Spain; mfuertes@neiker.eus (M.F.); agarcia@neiker.eus (A.L.G.-P.); 4 Area de Sistemas de Producción Animal, Servicio Regional de Investigación y Desarrollo Agroalimentario, SERIDA, E-33300 Villaviciosa, Asturias, Spain; albeitar@centroveterinarioalbeitar.com

**Keywords:** facial eczema, sheep, pathology, pithomycotoxicosis, immunohistochemistry, cirrhosis, cholestasis, liver

## Abstract

**Simple Summary:**

Facial eczema (FE) is a secondary photosensitization disease of farm ruminants caused by the sporidesmin A, present in the spores of the saprophytic fungus *Pithomyces chartarum*. This study communicates an outbreak of ovine FE in Asturias (Spain) and characterizes the local immune response that may contribute to liver damage promoting cholestasis and progression towards fibrosis and cirrhosis. Animals showed clinical signs of photosensitivity and lower gain of weight, loss of wool and crusting in the head for at least 6 months after the FE outbreak. Some sheep presented acute lesions characterized by subcutaneous edema in the head, cholestasis and nephrosis with macrophages and neutrophils present in areas of canalicular cholestasis. In chronic cases, alopecia and crusting, hepatic atrophy with regenerative nodules, fibrosis and gallstones were seen. The surviving parenchyma persisted with a jigsaw pattern characteristic of biliary cirrhosis. Concentric and eccentric myointimal proliferation was found in arteries near damaged bile ducts, where macrophages and lymphocytes were also observed.

**Abstract:**

Facial eczema (FE) is a secondary photosensitization disease of farm ruminants caused by the sporidesmin A, produced in the spores of the saprophytic fungus *Pithomyces chartarum*. This study communicates an outbreak of ovine FE in Asturias (Spain) and characterizes the serum biochemical pattern and the immune response that may contribute to liver damage, favoring cholestasis and the progression to fibrosis and cirrhosis. Animals showed clinical signs of photosensitivity, with decrease of daily weight gain and loss of wool and crusting for at least 6 months after the FE outbreak. Serum activity of γ-glutamyltransferase and alkaline phosphatase were significantly increased in sheep with skin lesions. In the acute phase, edematous skin lesions in the head, hepatocytic and canalicular cholestasis in centrilobular regions, presence of neutrophils in small clumps surrounding deposits of bile pigment, ductular proliferation, as well as cholemic nephrosis, were observed. Macrophages, stained positively for MAC387, were found in areas of canalicular cholestasis. In the chronic phase, areas of alopecia and crusting were seen in the head, and the liver was atrophic with large regeneration nodules and gallstones. Fibrosis around dilated bile ducts, “typical” and “atypical” ductular reaction and an inflammatory infiltrate composed of lymphocytes and pigmented macrophages, with iron deposits and lipofuscin, were found. The surviving parenchyma persisted with a jigsaw pattern characteristic of biliary cirrhosis. Concentric and eccentric myointimal proliferation was found in arteries near damaged bile ducts. In cirrhotic livers, stellated cells, ductular reaction, ectatic bile ducts and presence of M2 macrophages and lymphocytes, were observed in areas of bile ductular reaction.

## 1. Introduction

Facial eczema (pithomycotoxicosis) (FE) is a secondary-hepatogenous photosensitization disease of farm ruminants caused by the epipolythiodioxopiperazine mycotoxin sporidesmin A, present in the spores of the saprophytic fungus *Pithomyces chartarum* [1,2]. This fungus grows on dead vegetable matter at the base of the ryegrass-dominant pasture in all temperate worldwide zones during cloudy days with rain, with temperatures above 16 °C (optimal 24 °C), and relative humidity upper than 80% [3]. The portal circulation is the main route by which sporidesmin A enters to the liver following absorption from the intestinal tract [4]. In the biliary system, the sporidesmin A, characterized by the presence of an internal disulphide bridge, leads to the formation of toxic free-radicals that react with molecular oxygen to produce superoxide radicals [2,5,6]. The damaged liver is unable to remove a normal end product of chlorophyll metabolism, phylloerythrin (a photodynamic agent), from the blood for excretion in the bile [5]. When ruminants are exposed to sunlight, this pigment is responsible for unpigmented skin lesions [7]. FE was first recognized in New Zealand where it occurs more frequently [7]. However, this mycotoxic disease has also been reported in South Africa [8], Australia [9], the United States [10], France [11], Portugal [12], the Netherlands [13], Turkey [14], Uruguay and Argentina [3]. In Spain, the first and only reported outbreak occurred in the Basque Country [15]. In live animals, increases in the serum concentration of several enzymes such as gamma-glutamyltransferase (GGT) were found to be positively correlated to cholestasis and are indicators of bile duct damage in sporidesmin natural and experimental intoxication [1,7,12,16,17,18,19].

FE commonly affects sheep and cattle [7] while goats are more resistant to sporidesmin toxicosis than sheep [1]. The experimental sporidesmin toxicity in the rabbits has been demonstrated [17]. Although there was variation in susceptibility between individuals, the degree of liver injury and photosensitization appears to increase with both dose and length of time during which sporidesmin was administered [20]. The toxicity of pastures depends on the number of *P. chartarum* spores in the dead plant material and the toxicity of the particular *P. chartarum* strains [3]. Some authors reported that the most severe liver injury was due to the higher total consumption of spores caused by the combination of pasture spore concentration and dry matter intake [21]. Field observations reported that high spore counts (more than 40,000 spores/g of grass) of *P. chartarum* in grass samples can cause clinical signs [12], and there was a strong relationship between spore counts in ruminal content and severity of clinical signs [14]. In sheep, the clinical signs of photosensitization (erythema, edema and alopecia in unpigmented skin) appeared 14–18 days after intake of the toxin [1,20]. Jaundice [3] and loss of body weight in severe and chronic FE [7,20] are evident in this disease. In acute cases the liver is enlarged and shows a yellowish discoloration [10], and the gallbladder and extrahepatic bile ducts are distended [17]. In cases of longer evolution, there is liver atrophy and fibrosis [3] leading to liver cirrhosis [8,15].

The histological lesions include acute necrotizing cholangitis [12,17], bile stasis [1] and later on, bile duct hyperplasia and portal fibrosis [3,10,15]. Liver regeneration [7] and interlobular cirrhosis [8] have also been shown in the chronic phase of FE.

A lymphocytic infiltrate has been observed associated with the chronic hepatic changes of FE [8,10]. The main cell target in cholangiopathies are the epithelial cells lining the bile ducts (i.e., cholangiocytes), that are exposed to cytokines and inflammatory mediators produced by infiltrating lymphocytes, macrophages and activated myofibroblasts [22]. “Typical cholangiocyte proliferation” is usually found in acute obstructive cholestatic liver disease and “atypical ductular reaction” is commonly seen in human primary biliary cirrhosis [23]. Bile duct lesions have been described in subacute FE in cattle [24], but there are no studies about the type of ductular response in chronic FE cases or on how ductular cells interact with other cell types such as Kupffer and hepatic stellate cells (HSCs). Quiescent HSCs are resident perisinusoidal cells in the subendothelial space between hepatocytes and sinusoidal endothelial cells [25]. They are the primary site for storing retinoids (vitamin A) within the body. During liver injury HSCs proliferate and differentiate into contractile and matrix-producing myofibroblasts that generate progressive fibrosis and promoted a chemotactic activity for monocytes and lymphocytes, among others [25,26,27]. In liver pathology, ample evidence has been provided for an indirect role of macrophages in the development of fibrosis [25].

The main objective of this study is to characterize the inflammatory infiltrate, as well as the presence of HSCs and their distribution in damaged hepatic tissue in natural cases of FE from an outbreak that occurred in Northwestern Spain. Additionally, the acute and chronic liver lesions found in these cases of the disease, with special reference to the ductular reaction, are characterized, mainly considering their relationship with the presence of inflammatory cells, as a possible indicator of the role that the immune response can play in the development of liver damage in this toxicosis.

## 2. Materials and Methods

### 2.1. Ethical Information

Experimental animals were not used in this work. An observational study was performed with the blood samples obtained during regular veterinary clinical services and with *post mortem* tissue samples that are routinely collected after the death of animals.

### 2.2. Case History and Clinical Observations

The outbreak of FE occurred in La Mata, Grado, an inland municipality in the Principality of Asturias, in the north-west of Spain, between the end of September and the beginning of October 2003. This estate belonged to Servicio Regional de Investigación y Desarrollo Agroalimentario (SERIDA), Asturias. According to official data of the Asturian Society of Economic and Industrial Studies and the Meteorological Territorial Center of Asturias, the summer climate of 2003 was dry and very warm, with temperatures higher than 30 °C in the inland [28]. The autumn was warm (with temperatures above 19 °C in September and the first 19 days of October) and very rainy with a total monthly rainfall of 141.4 and 219 L/m^2^ in October and November, respectively. The affected sheep were grazing on two plots (named 1B and “mixtures”) at 50 m altitude. The plot 1B, of a 3.2 hectare (ha) of land, was sowed in 2001 with perennial ryegrass (*Lolium perenne* L. var. Tove), 30 kg/ha; hybrid ryegrass (*Lolium boucheanum* var. Kunth), 12 kg/ha and white clover (*Trifolium repens* var. Huia), 3 kg/ha. The plot mixtures of 2.5 ha was sowed in 1993 with perennial ryegrass (*Lolium perenne* L. var. Phoenix), hybrid ryegrass (*Lolium boucheanum* variety Dalita), and white clover (*Trifolium repens* var. Huia), at the same doses. In the plot 1B 28 crossbreed female sheep and 11 ewe lambs were grazing, of which 7 (6 adult and 1 ewe lamb) showed clinical signs of photosensitivity (pruritus, erythema and alopecia) on the face and ears. In the plot mixtures 2 adult sheep (over a total of 24) and 3 ewe lambs (over a total of 11) developed similar clinical signs, according to the information supplied by the practitioner. The four most affected animals had been treated symptomatically with Alergia-N (Pfizer), an antihistamine drug (cyprohetadine chlorhydrate, chlorphenamine maleate), and Penbex (Industrial veterinaria, S.A-INVESA), for treatment of secondary bacterial infections caused by germs sensitive to the association penicillin-dihydrostreptomycin sulfate, both by intramuscular route. Initially, the face skin lesions recovered when the animals were removed from the pastures for some days into the shade, but began to appear when the sheep returned to the plots and were exposed to sunlight. In the following 6 months, animals involved in the FE outbreak 4 sheep and 1 ewe lamb (plot 1B) and 1 sheep and 2 ewe lambs (plot mixtures) showed lower weight gains, wool loss and crusting in the dorsum of the head, nose and ears were observed.

### 2.3. Biochemical Assay and Statistical Analysis

Blood serum samples were taken from the jugular vein from 73 sheep (11 with and 62 without skin lesions) during the chronic stage of the episode (six months after the FE outbreak). Serum concentrations of γ-glutamyltransferase (GGT), alkaline phosphatase (ALP), aspartate aminotransferase (AST), albumin and total protein (TP) were determined on a multianalyser (Cobas Integra 400, Roche diagnostics). The results of the serum parameters analyzed were reported as mean, standard deviations and range (minimum and maximum), calculated using routine descriptive statistical procedures. The Kolmogorov–Smirnov test was used to assess normality of data. Non-parametric statistical methods were used to compare groups. Mann-Whitney U test was employed to compare the exposed animals that not showed cutaneous clinical signs with exposed sheep that presented skin lesions. *p*-values of less than 0.05 were considered statistically significant. All the statistical analyses were performed with the R software version 3.6.1 (R Development Core Team, R Foundation for Statistical Computing, Vienna, Austria, December 2019).

### 2.4. P. chartarum Spore Counts

A total of 36 grass samples from every field were randomly collected to count *P. chartarum* spores/g on 23 November 2003. Each grass sample was taken at least 10 m apart. The grass was cut 1 cm above the ground, avoiding taking soil, and cut into pieces of approximately 4 cm in length. From this mixture, 15 g of grass was taken and 150 mL of water was added, and then the mixture was homogenized for 3 min, in order to release the spores in the water. The *P. chartarum* spores present in each sample were identified and counted in an aliquot of wash water, using a Fusch-Rossental chamber. Finally, depending on the volume of water investigated, calculations were made to express the results as number of spores/g of grass. These grass samples were analysed in the Department of Animal Health of NEIKER- Basque Institute for Agricultural Research and Development.

### 2.5. Animal Cases and Pathological Examination

One adult sheep (plot 1B) with clinical signs suffering the acute phase of the disease, two adult animals (1 of the plot 1B and 1 of the plot mixtures), two months after the FE outbreak, and two adult sheep of the plot 1B six months after the FE outbreak, all of them belonging to the Galician breed, were examined in this study. Two sheep of a different estate were selected as reference and healthy animals. All of them were submitted to the Pathologic Diagnostic Service of the Veterinary Faculty of León over a seven-month period (November 2003–May 2004). In November an adult sheep with clinical signs in the acute phase of FE was humanely euthanized in the flock and were submitted for necropsy. Only when the pathological findings were discussed with the clinician, there was enough evidence to consider sporidesmin toxicosis as a possible cause of the liver and skin lesions. Two affected sheep and two other healthy control sheep were examined at slaughter in January and different tissue specimens (liver, skin, kidney, lung, heart) were submitted for histopathology. Finally, in May, two adult alive sheep were submitted for necropsy and euthanasia was performed by intravenously injection of barbiturate (T61; Intervet International, Madrid, Spain), after xylazine (Bayer, Leverkusen, Germany) subcutaneous administration, followed by exsanguination after the severing of carotid arteries and jugular veins according to our institution guidelines. Complete necropsies were performed in 3 sheep, one submitted during the acute, and two during chronic phases of FE. After gross examination, representative tissue samples (skin, liver, kidney, spleen, mesenteric lymph nodes, intestine, pancreas, adrenal glands, heart, lung, skeletal muscle and brain), were collected in all necropsied animals. All samples were fixed in 10% neutral-buffered formalin, processed routinely and embedded in paraffin wax. Sections (4 µm) were cut, mounted on glass microscope slides and stained with haematoxylin and eosin (HE), Masson Goldner trichrome for collagen, AFIP (Armed Forces Institute of Pathology) method for lipofuscin, Hall´s bilirubin stain, Perls´ Prussian blue stain for ferric pigments and acid rubeanic method for copper. Two livers with lesions consistent with steatosis and macronodular cirrhosis belonging to sheep of plot 1B that died in January and May, respectively, were not included in this study due to severe *post mortem* alterations (autolysis and putrefaction).

### 2.6. Immunohistochemistry

Selected sections (4 µm) from the liver were immunohistochemically labelled with a panel of antibodies. In all the cases, a polymer-based detection system (EnVision^®^ System Labelled Polymer-HRP; Dako, Glostrup, Denmark) was employed, following the manufacturer instructions. Subsequently, immunolabelling was developed with a solution of 3.3´diaminobenzidine (DAB) or AP solution (Vector Laboratories, Burlingame, CA, USA). The slides were counterstained with Mayer’s haematoxylin and mounted in hydrophobic medium (Table 1).

## 3. Results

### 3.1. Serum Biochemistry

Table 2 showed the mean ± standard deviation and range (minimum-maximum) of albumin, TP, AST, ALP and GGT concentrations according to the clinical status: exposed animals that not showed cutaneous clinical signs (a) and exposed sheep that presented skin lesions (b). Serum chemistry references values in sheep are shown in Table 2 for each biochemical parameter analyzed (c) [29].

### 3.2. P. chartarum Spore Counts

Mean ± standard deviation and range (minimum-maximum) of the number of *P. chartarum* spores per gram of pasture in the 36 grass samples analyzed, was 11,389 ± 15,100 (0–75,000) spores/g of grass.

### 3.3. Pathology

#### 3.3.1. Acute Stage

**Figure 1:**
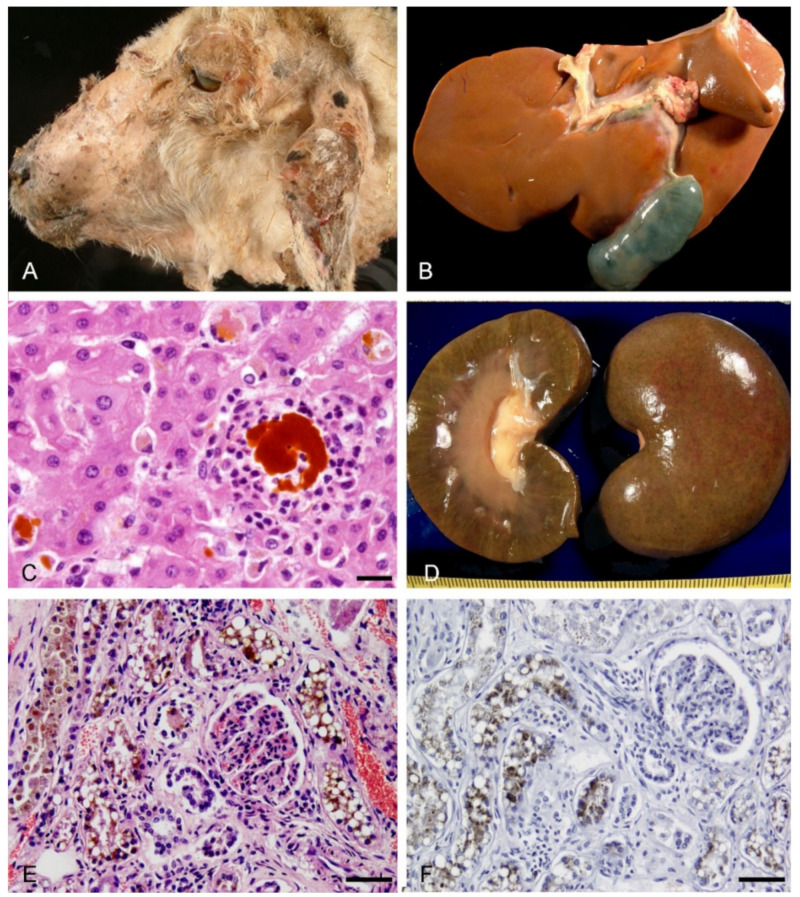
Acute lesions observed in naturally acquired cases of FE in sheep. (**A**) Sloughing of the skin in the ears, eyelids, muzzle and lips. (**B**) The liver was diffusely yellowish in color and the common and cystic bile duct were distended. (**C**) Polimorphonuclear neutrophils were surrounding a hepatic “bile lake”. HE. Bar, 20 µm. (**D**) Linear greenish discoloration of the renal cortex and medulla. (**E**) Brown granular pigment in the proximal tubules. HE. Bar, 50 µm. (**F**) Green granular pigment consistent with bilirubin in the proximal tubules. Hall´s bilirubin stain. Bar, 50 µm.

Animals examined at this stage of the disease showed edematous drooping ears, serum exudation and sloughing of the skin in the dorsum of the ears, eyelids, muzzle, lips and forelimbs, characterized histologically by epidermal necrosis and presence of serocellular crusts (Figure 1A). The carcass showed intense jaundice and the liver were bile stained (yellowish) and enlarged (Figure 1B). The extrahepatic bile ducts and the gallbladder were distended by abundant bile and visible brown concretions (pigmented calculi) (Figure 1B). Histologically, in the liver, there was a macrovesicular (large droplet) predominantly periportal fatty change (zone 1). Hepatocytic and canalicular cholestasis with bile granular deposits in the cytoplasm of hepatocytes and presence of bile plugs in canaliculi respectively, confirmed by the Hall histochemical stain, were prominent in centrilobular regions (zone 3). Some of these cells were swollen or necrotic. Small clumps of polimorphonuclear neutrophils (PMNs), Kupffer cells (KCs) with AFIP lipofuscin positive granules, and foreign-body-type giant cells were present surrounding more prominent deposits of bile pigment (‘bile lakes’) (Figure 1C). Portal tracts were edematous with ductular proliferation at the periphery (bile ducts with a well-defined lumen). There was also a mild (sparse) portal inflammation (scattered lipofuscin-laden macrophages, detected by the AFIP method, and lymphocytes). The epithelial cells in septal bile ducts were shrunken with picnotic nuclei, and larger bile ducts were dilated and contained inspissated bile. The kidney was macroscopically slightly enlarged and showed a greenish discoloration with linear green streaks throughout the cortex and medulla (Figure 1D). Diffuse cellular swelling and green granular pigmented bilirubin, in the proximal tubules, as well as green-yellow acellular tubular bile casts in the distal nephron segments of the renal medulla, both confirmed by the Hall histochemical stain, were observed microscopically (cholemic nephrosis) (Figure 1E,F).

#### 3.3.2. Chronic Stage

**Figure 2:**
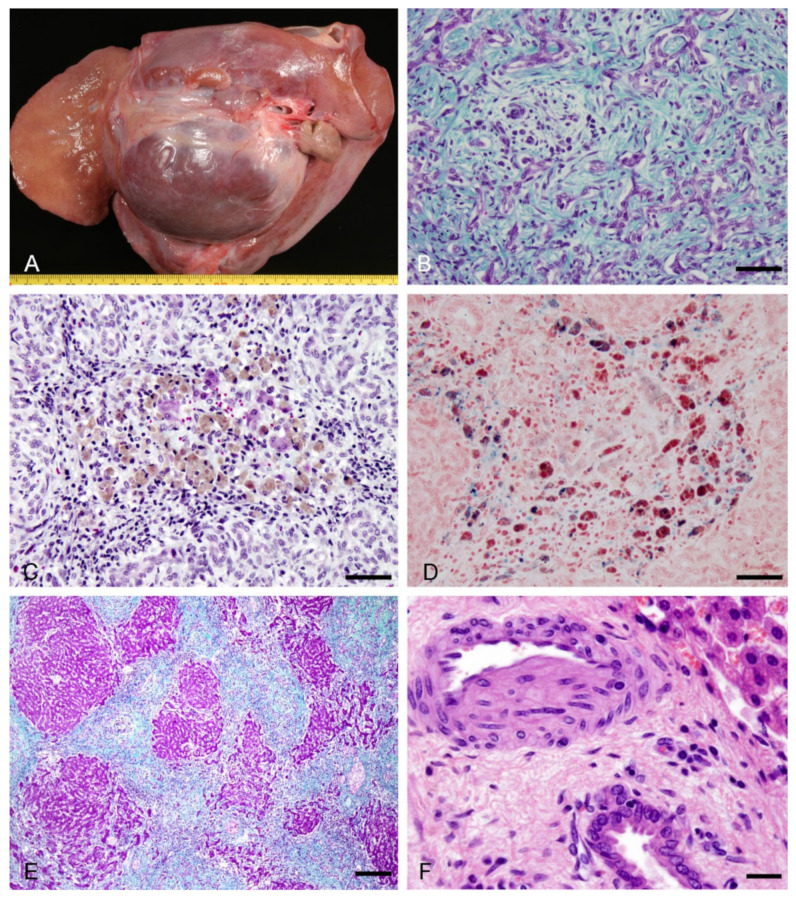
Chronic lesions observed in naturally acquired cases of FE in sheep. (**A**) Marked atrophy in the hepatic left lobe and a large nodule in the hepatic visceral surface. (**B**) Hepatic lobular areas are replaced by proliferated bile ductules (atypical ductular reaction) and fibrous tissue. Masson-Goldner trichome stain. Bar, 50 µm. (**C**) Numerous lymphocytes and pigmented macrophages were seen in the fibrous tissue in association with remnants of hepatic lobes. Masson-Goldner trichome stain. Bar, 50 µm. (**D**) Macrophages were positively red stained for lipofuscin (AFIP stain) that coexisted in some cells with hemosiderin. Perls’ Prussian blue stain. Bar, 50 µm. (**E**) Jigsaw pattern characteristic of the biliary cirrhosis. Masson-Goldner trichome stain. Bar, 200 µm. (**F**) Eccentric myointimal proliferation in a hepatic arteriole adjacent to the bile duct. HE. Bar, 20 µm.

All the sheep submitted 2 and 6 months after the initial onset of the disease outbreak showed skin lesions limited to areas of the head: dorsum of the head and ears, eyelids, face, lips and nose were alopecic and crusting. In the ears, there was focal necrosis of the epidermis and the underlying cartilage with palisading crust formation. The livers were atrophic, with lesions most marked in the left lobe (2 sheep), yellowish with whitish bands of fibrous tissue and larger lonely nodules of 4 and 8 cm located principally in the visceral surface in the quadrate lobe (2 sheep) (Figure 2A). The intrahepatic and extrahepatic bile ducts and gallbladder were also enlarged and contained biliary sludge and pigmented gallstones.

Histologically, increased biliary fibrosis with an associated inflammatory infiltrate, and prominent ductular proliferation were observed in the liver of all sheep examined. The portal tracts were expanded with proliferating bile ductules with a well-defined lumen (typical cholangiocyte proliferation) and fibrous tissue, which occasionally bridged adjacent portal tracts. Extensive pericellular or subsinusoidal fibrosis was evident using Masson-Goldner trichrome stain. An intense leucocytic portal inflammation (lymphocytes and plasma cells) was noted. At the same time and in the same liver, areas formed by an irregular proliferation of intrahepatic bile ductules at the portal tract margins, with poorly formed lumina that replaces the hepatic parenchyma (atypical ductular reaction) (Figure 2B). Numerous spindle-shaped cells, lymphocytes and macrophages containing brown pigment, were seen throughout the fibrous tissue, as well as in remnants of the hepatic lobules which eventually disappeared, principally in the hepatic left lobe (Figure 2C).

Histochemical studies showed that pigmented macrophages in close association with remnants of hepatocytes were positively red-stained with AFIP method for lipofuscin. This pigmented lipoproteins coexisted in some macrophages with iron deposits (hemosiderin) that reacted with Perls´ Prussian blue stain (Figure 2D). Granular deposits of protein-bound copper salts were observed in periportal hepatocytes and pigmented macrophages as small black granules in their cytoplasm. Collagen fibers were arranged in concentric layers around dilated interlobular bile ducts and proliferating bile ductules (“onion skin” fibrosis). As the result of coalescence of adjacent fibrotic portal tracts, portal-portal fibrous septa were noticed and the surviving parenchyma persisted with a jigsaw pattern characteristic of the biliary cirrhosis (Figure 2E). Larger bile ducts in sheep examined two months after FE outbreak were dilated and contained inspissated bile plugs and biliary stones. The biliary epithelium was flattened and necrotic and the fibrotic wall contained numerous pigmented macrophages and foreign body type giant cells around bile pigment deposits, admixed with mononuclear inflammatory cells. A marked fibrotic thickening, with a mild lymphocyte infiltrate, was the major histologic finding in large intrahepatic and extrahepatic bile ducts 6 months after the FE outbreak. Canalicular cholestatic changes were only seen in one sheep 2 months after the outbreak. Additionally, this sheep with cholestasis also showed brownish discoloration of the renal cortex and medulla. Intracellular bile pigment, stained green by the Hall histochemical stain, involved proximal tubules in two sheep 2 months after the FE outbreak (cholemic nephrosis).

All sheep examined in the chronic stage showed vascular lesions in the liver. In portal hepatic arteries, hepatic arteries in the septa and large hilar hepatic arteries, near severely damaged intrahepatic (interlobular, septal) and extrahepatic (common and cystic) bile ducts, both concentric and eccentric myointimal proliferation was found to some degree (Figure 2F). Similar occlusive lesions, characterized by intimal cap proliferation, were observed in sublobular veins near damaged bile ducts. Lymphocytic phlebitis and phlebosclerosis consisting, respectively, of chronic inflammatory infiltrate of the wall and perivenular fibrous thickening of central veins and striking fibromuscular hypertrophy of the walls of ectatic hepatic veins were also found.

### 3.4. Immunohistochemistry

**Figure 3:**
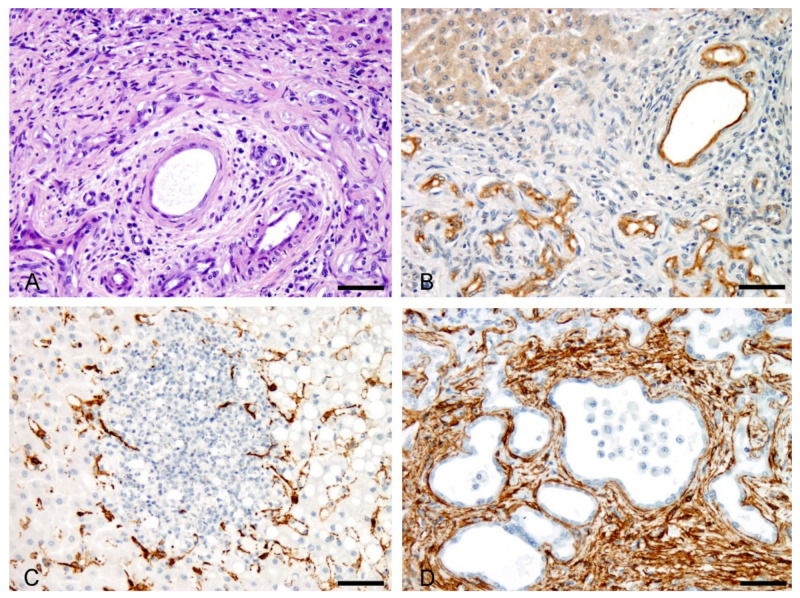
Immunohistochemistry in naturally acute and chronic acquired cases of FE in sheep. (**A**) Ectatic interlobular bile duct surrounded by a thin layer of collagen fibers (‘onion skin fibrosis’). HE. Bar, 50 µm. (**B**) Pancytokeratin antibody stain positively ectatic bile ducts with flattened epthelium, similar to showed in Figure 3A, and bile ductules. Bar, 50 µm. (**C**) α-SMA-+ HSCs in areas of canalicular cholestasis in acute FE liver lesions. Bar, 50 µm. (**D**) In chronic liver lesions observed in FE, α-SMA-+ HSCs cells accumulated and surrounded ectatic bile ducts. Bar, 50 µm.

The epithelial cell marker (pan-cytokeratin) was strongly expressed in the epithelium of bile ducts and, with less intensity, in acute and chronic biliary ductular reaction. This antibody was especially useful for the identification of the bile duct epithelium of damaged bile ducts that developed granulomas composed of macrophages, as well as the ectatic bile ducts surrounded by fibrous tissue in portal tracts (Figure 3A,B).

**Figure 4:**
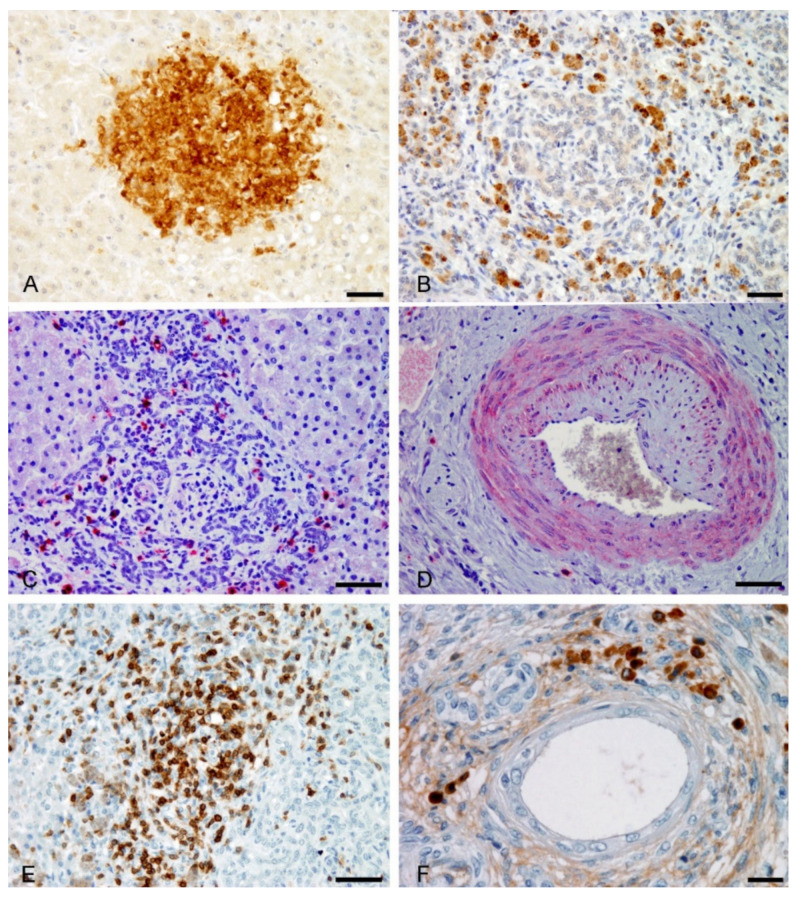
Immunohistochemistry in naturally acute and chronic acquired cases of FE in sheep. (**A**) Intense positive MAC387 immunolabeling of a cluster of macrophages and neutrophils in an area of cholestasis (acute lesion). Bar, 50 µm. (**B**) Presence of CD206-+ macrophages in areas of fibrosis and ductular reaction (DR) (chronic lesion). Bar, 50 µm. (**C**) Anti TGF-β antibody red stained cells morphologically compatible with macrophages in areas of DR (chronic lesion). Bar, 50 µm. (**D**) Positive immunolabeling for anti TGF-β antibody in a hepatic artery with an occlusive lesion (subintimal proliferation) in chronic FE cases. Bar, 50 µm. (**E**) Aggregates of T CD3-+ lymphocytes in areas of DR in chronic FE lesions. Bar, 50 µm. (**F**) In chronic FE lesions IgG-+ plasma cells were seen scattered around ectatic bile ducts. Bar, 20 µm.

In the acute phase, there was an increase of α-SMA-positive into the parenchyma in areas of canalicular cholestasis and hepatocyte necrosis as well as within portal tracts around proliferating typical bile ductules (Figure 3C). In chronic FE these cells showed strong immunopositivity and accumulate surrounding bile ductular structures and ectatic bile ducts in fibrotic portal tracts and septa (Figure 3D). In this last case α-SMA-positively immunolabelled HSCs, appeared as spindle-shaped cells that delimited both bile ducts and atypical ductules. α-SMA was not expressed in ductular cells, cholangyocytes or hepatocytes. An increase in the number of macrophages and PMNs that stained positively for both anti-lysozyme and anti-MAC387 antibodies was observed in the areas of canalicular cholestasis and hepatocyte necrosis in acute FE liver lesions (Figure 4A). Many cells with the characteristic morphology of KCs have strong cytoplasmic labelling for lysozyme, and MAC387 immunostaining clearly defined cell aggregates, with extensive immunoreactivity, scattered in the liver parenchyma, both in zones 2 and 3 of the hepatic lobes. In addition MAC387 positivity was observed in cells having the morphology of blood monocytes within blood vessels.

In the chronic hepatic lesions, abundant pigmented macrophages in close association with remnants of hepatocyte lobules, were positive for lysozyme and MAC387. Also, strong lysozyme and MAC387 positively immunolabelled macrophages were found as part of the granulomatous lesions formed around degenerated bile ducts. Remarkably, a decrease in the number of cells immunostained with these antibodies were observed in the fibrous septa in relation to atypical ductular proliferation. The mannose receptor (CD206) staining was only present in some pigmented macrophages observed as scattered cells in the portal tracts (acute phase), but were more numerous in chronic lesions, in fibrotic septa with marked ductular reaction (Figure 4B). No co-localization was found with lysozyme and MAC387 markers within cells in the hepatic lobes. It is remarkable that cells positively immunostained for CD206 antibody corresponded to macrophages showing intense TGF-β immunoreactivity. (Figure 4C). Sparse to intense intracellular staining for TGF- β was also observed in α-SMA-positive cells present in the thickened subendothelial areas and in the tunica media of hepatic arteries (Figure 4D).

In acute FE lesions, intrahepatic T CD3+-lymphocytes were scattered in sinusoids and portal tracts and occasionally were seen in intraepithelial location in bile ducts. In chronic FE lesions, there was a prominent T lymphocytic infiltrate forming aggregates intimately associated with the bile ducts, with a diffuse pattern or forming aggregates in areas of bile ductular reaction in the fibrous septa (Figure 4E). T CD3-positively immunostained lymphocytes were identified in granulomas around degenerate bile ducts and extravasated bile pigment as well as surrounding large bile ducts contain bile stones. IgG+ plasma cells were occasionally seen in portal tracts in acute FE liver lesions. In chronic cases, plasma cells were found scattered or in small amounts followed a similar distributional pattern than T lymphocytes, although they were less abundant. Like T lympohocytes, plasma cells were found in the concentric arrangement of fibrous tissue around bile ducts and ductules (Figure 4F).

## 4. Discussion

In this report we described an episode of FE in sheep that occurred in Asturias, Spain in 2003. This mycotoxic disease was first observed in European sheep in France [11] and later in the Basque Country, Spain [15] and the Azores, Portugal [12]. In late 2003, the weather conditions in Asturias were the most favorable for *P. chartarum* growth and sporulation. The autumn was warm (with temperatures above 19 °C in September and the first 19 days of October) and very rainy, with total monthly rainfall of 141.4 and 219 L/m^2^ in October and November, respectively. Late summer and early autumn with warm temperatures (minimum 16 °C) and high humidity (above 90 per cent) were the periods favorable to FE outbreaks in the Azores, Portugal between 1999 to 2001 [12]. The periods and weather conditions in Asturias were similar to those observed in these islands, both with oceanic climate. Spore counts of *P. chartarum* identified in several different grass samples ranged from 0 to 75,000 spores/g of pasture. It has been suggested that spore counts as low as 50,000 spores/g of grass could be dangerous to livestock if grazed for long periods in sunlight and that the greater liver injury occurred when sheep were grazed on pastures having maximum spore counts of 130,000 spores/g of grass [20,21]. Other causes of toxins from fungi or plants were not found around the studied field. Given that FE clinical signs appear 14–18 days after intake of the mycotoxin [20] and, in the present outbreak, sheep with acute signs of photosensitization were observed from the middle of October, plots possibly reached the higher spore numbers in September/October. The low spore counts observed in the present study may be related to the variability between individual sites in the plot. Besides, the grass samples were collected in late November with a low temperature (average of 11.6 °C), conditions less favorable to fungal growth and sporulation.

In the present study, a significant rise in the activity of serum GGT associated with histological cholestasis and bile duct damage was demonstrated in sheep in accordance with previous reports in which sporidesmin was administered experimentally [16,30], and also in spontaneous intoxications [10,12,14,15]. GGT and ALP levels were higher in all sheep examined (when compared with reference ranges) and significantly increased (*p* < 0.001 and *p* < 0.01, respectively) in the 11 sheep with skin lesions when comparing with the 62 apparently healthy animals (without skin lesions). In our opinion, in agreement with previous reports [7,18], the detection of elevated GGT serum levels was the most suitable marker for monitoring FE affected sheep under field conditions because it correlated with the liver lesions, even in apparently healthy animals. In this sense, our results confirmed that a high activity of GGT persists for six months after an FE outbreak, in accordance with other published data [3]. Serum albumin was lower (*p* < 0.01) and TP was increased (*p* < 0.05) in sheep with skin lesions and the serum AST levels did not show statistically significant changes. AST was increased in all the tested animals, even in those without injury 6 months after the outbreak. This fact could be the consequence of a previous exposure and the development of a chronic inflammatory process, so that AST could be a sensitive and long-lived marker of liver damage in sheep, in disagreement with previous observations of natural intoxication with sporidesmin in sheep [18].

The gross lesions in the skin, liver and kidney described in the acute phase of ovine FE agree, in general, with previous descriptions in natural [10,14] and experimental cases [8,30]. Microscopic lesions described in the acute stage have been demonstrated experimentally in sheep dosed pure cultures of *P. chartarum* directly to the stomach, equivalent to approximately 3–4 mg/kg live mass [8,30]. These animals, that became photosensitive on the 9–10th day and died or were euthanized on the 4–10 subsequent days, showed hepatic parenchymal infarcts with leakage of bile between the necrotic hepatocytes and the presence of polymorphonuclear cells, similar to the microscopic findings described in the present study. A moderate bile duct proliferation and mononuclear cell infiltration (including some pigment laden macrophages), as well as necrotic lesions in the bile ducts in the portal tracts, were also observed in this work. Recently, altered cell adhesion and disruption of actin in sheep gallbladder epithelial cells incubated with sporidesmin were demonstrated, suggesting that the biliary tract pathology in FE may be due to the effects of the toxin on cytoplasmic and cell surface protein networks, affecting the integrity of the epithelial lining of the biliary tract [31]. The special stains used, for the first time in the present study, such as Hall and AFIP stains, were useful to demonstrate the presence of bile and lipofuscin, respectively, in liver. Lipofuscin can accumulate in liver macrophages in natural FE in sheep and may represent the remnants of phagocytosed debris from necrotic hepatocytes. This study also confirmed by the Hall stain the presence of cholemic nephrosis characterized by proximal tubulopathy and intrarenal bile cast formation in ovine FE. Previous studies showed that elevated plasma levels of conjugated bilirubin are related to renal failure associated with obstructive jaundice [32]. The term “bile cast nephropathy”, caused by direct bilirubin toxicity and tubular obstruction, has been proposed for this pathologic entity in humans [33].

Liver atrophy, more severe in the left lobe, and large nodules of regeneration in the visceral surface of the liver, as well as alopecic and crusty head skin were the most striking gross findings in chronic forms of FE that are in agreement with previous descriptions [3,15,34].

The atrophic left hepatic lobe with dilated intrahepatic and extrahepatic bile ducts are conditions that may have been due to compression of the left trunk of the portal vein secondary to hepatolithiasis [35]. In our case, the mechanism of left lobar atrophy might have been due to the occlusion of the ducts by biliary sludge and pigmented gallstones that cause atrophy of the parenchyma served by them [34].

The presence of nodular regeneration and cirrhosis have been considered the most conspicuous pathological changes in natural cases of FE in sheep [8] and may start as early as 2 weeks after toxin insults [7]. Experimentally, this lesion has been observed in sheep challenged with a total of 2.125 mg/kg sporidesmin divided into 17 doses for 144 days [30].

A remarkable histological finding in chronic FE was a strong ductular reaction that replaces the hepatic parenchyma associated with extensive fibrosis and aggregates of lymphocytes and pigment containing macrophages in connective tissue septa. It has been suggested that this ductular reaction can represent regenerative proliferation of bipotential hepatic stem/progenitor cells that have the ability to differentiate into both hepatocytes and cholangiocytes, but there is no definitive evidence for it [23]. Pigmented macrophages contained deposits of lipofuscin, hemosiderin and copper salts, possibly as a result of an increase of their oxidative stress with iron-catalyzed production of reactive oxygen species causing oxidative damage to lipids and proteins [36]. In vitro, the autoxidation of reduced sporidesmin is catalyzed by iron and by copper and generate a dithiol, a superoxide free radical suggesting that any superoxide production from sporidesmin in vivo would be mediated by the intracellular transport pool of copper [37]. Lipofuscin and copper are also deposited in biliary cirrhosis and chronic cholestatic diseases, respectively [38].

In this study liver vascular lesions were constantly seen in the chronic phase of FE near affected bile ducts. It is known that extrahepatic and intrahepatic bile ducts are located with branches of the hepatic artery (their sole blood supply) and portal vein [39]. Eccentric subintimal fibroblastic proliferation on the side adjacent to affected bile ducts were sometimes seen in sheep and goats [1,8] and adult cows [24]. It has been suggested that a high concentration of sporidesmin injures the biliary epithelium and the release of toxin and bile acids produce irritative lesions and coagulative necrosis of blood vessels, both arteries and veins [34].

Ductular reaction (DR) appears to be one of the factors that deteriorate liver function, because gradually replaces the hepatic parenchyma and causes a gradual decrease in mature hepatocytes [40]. In this study DR was recognized as bile duct hyperplasia in extensive areas of the liver in chronic FE cases and the cells present in the lesion, immunostained positively for pancytokeratins but did not express mesenchymal cell markers [23]. DR is observed in cholestatic liver diseases and is closely related to liver fibrosis induced by HSCs and portal fibroblasts and is also an important factor for liver regeneration [41]. According to these last authors, the mechanism responsible for DR is not definitively understood and cholangiocytes, hepatocytes, or hepatic progenitor cells can be the origin of active cells during DR, depending on specific liver injury.

In FE cirrhotic livers the fibrous septa contain large numbers of HSCs expressing protein α-SMA. It is known that, as HSCs activate, the expression of α-SMA is increased, which confers contractile potential to the cells [25]. HSCs are the major source of type I collagen and other extracellular matrix proteins that characterize the fibrotic liver [25,42].

The sporidesmin elicits biliary insult and an activation of HSCs alongside induction of hepatic inflammation. In the acute exposure to this toxin, an inflammatory response of neutrophils, lysozyme and calprotectin in KCs was observed in areas of cholestasis. This fact could suggest that KC response occurs early in cholestatic injury and bile acids leakage from cholangioles may be involved in this proliferation [27]. Previous studies indicate that HSCs activation also promotes the recruitment of leukocytes in the early phase of liver injury [26]. In chronic FE these macrophages were seen mainly around degenerated bile ducts and, to a lesser extent, in relation to DR. Nevertheless CD206+ macrophages were more numerous in this last location. These results suggest that in the acute and chronic phases of FE, the peribiliar inflammatory infiltrate is dominated by classically activated M1 macrophages. As a detail, in the chronic phase there was an increase of activated M2 macrophages associated with increased fibrogenesis [43]. These data indicated that M1 macrophages prevail during the onset of liver injury, and M2 macrophages, if liver injury becomes chronic, take up a profibrotic role secreting TGF-β. In this work TGF-β expression was observed in cells consistent with macrophages in fibrotic septa in areas of DR. There is evidence supporting an indirect role of M2 macrophages in the development of fibrosis secreting factors like TGF-β, which activate HSCs [44,45]. Besides this, in the present work, TGF-β immunostaining was observed in hepatic arterial vessels with vascular occlusive lesion. This cytokine plays a prominent role in vascular disorders such as the arterial thickening associated with pulmonary hypertension [46] and in other arterial pathologies, with effects on the changes of the vascular smooth-muscle cells during transition from structural to a synthetic phenotype [47]. It has been also documented that both M1 and M2 macrophages accumulate in fibrotic septa of mouse and human end-stage cirrhotic livers, suggesting that both are necessary in fibrotic responses [45]. An interesting finding in the hepatic immune response in the case of FE was the presence of numerous T CD3+ lymphocytes in proximity to HSCs in the fibrous septa and around damaged bile ducts in chronic FE lesions. There is evidence that HSCs secrete cytokines, such as TGF-β, that has lymphocyte chemotactic activity and contributes to recruit and positioning of lymphocytes within the liver stroma in order to maintain an effective immune response [26]. In addition, it has been suggested that T lymphocytes can interact with HSCs and secrete various cytokines to modulate and sustaining fibrotic responses in chronic liver disease [26,48]. In chronic FE sheep an accumulation of CD3+ lymphocytes, and few plasma cells expressing IgG were observed, similarly to alpha-naphthylisothiocyanate (ANIT)-induced biliary pathology in mice and other cholestatic liver diseases in humans [49]. The vascular and ductal system and its macroscopic shape of sheep’s liver are very similar to the human organ, so it has a great potential as an animal model [50].

## 5. Conclusions

FE eczema causes loss of weight, skin lesions and liver damage principally. The biochemical parameters of the blood samples suggest that GGT and AST are elevated in animals affected. Lesions such as liver atrophy and cirrhosis are characterized by a ductular reaction with the associated presence of HSCs, KCs, lymphocytes and neutrophils. These features vary depending on the time of previous exposure to the toxin, with a pro-inflammatory character in the acute phases and an anti-inflammatory, and therefore profibrotic, component in the chronic cases. T-lymphocyte infiltrates persist in chronic forms, in association with increased levels of HSCs, which could contribute to recruit lymphocytes within the liver stroma in order to maintain an effective immune response. Considering that the vascular and ductal system of the ovine liver have strong similarities to the human organ, sheep have remarkable potential as a suitable animal model to study the pathogenesis and the interaction of toxicosis that result in liver damage.

## Figures and Tables

**Table 1:** Antibodies, specificity and immunohistochemical procedure used.

Antibody	Clone	Type	Marker For	Antigen Retrieval	Dilution	Source
CD3	-	Rabbit, Policlonal	T cells	PTLink/pH6/20’	1:300	Dako, Denmark
IgG	-	Biotinylated antibody	Plasma cells	PTLink/pH9/20’	1:200	Vector Lab, USA
Calprotectin	MAC387	Mouse, Monoclonal	Macrophages, activated epithelial cells	PTLink/pH9/20’	1:200	Gene Tex, USA
Lysozyme	-	Rabbit, Policlonal	Macrophages	PTLink/pH6/20’	1:250	Dako, Denmark
CD 206	MR5D3	Rat, Monoclonal	Macrophages (mannose receptor)	PTLink/pH6/20’	1:100	Gene Tex, USA
TGF β	TGFB-1	Mouse, Monoclonal	Hepatic stellate cells, Kupffer cells	PTLink/pH6/20’	1:200	GeneTex, USA
α-SMA	1A4	Mouse, Monoclonal	Smooth muscle cells, Myofibroblasts, HSCs	PTLink/pH9/20’	1:100	Dako, Denmark
Pankeratin	Pck26	Mouse, Monoclonal	Epithelial cells	Trypsin, 15’	1:200	Dako, Denmark

**Table 2:** Serum liver enzymes in sheep with and without cutaneous lesions 6 months after the onset of the facial eczema (FE) outbreak. Different superscripts between columns shown significant differences between the animal groups compared (* *p* < 0.05; ** *p* < 0.01; *** *p* < 0.001).

	Albumin (g/dL)	TP (g/dL)	AST (U/L)	ALP (U/L)	GGT (U/L)
**(a) No Lesion (*n* = 62)**	3.72 ± 0.54 ^a^ ** (1.69–3.86)	7.20 ± 0.80 ^a^ * (4.81–9.39)	237.82 ± 142.76 ^a^ (89.7–762.6)	283.39 ± 180.06 ^a^ ** (34–954.9)	156.15 ± 266.28 ^a^ *** (40.1–1838.30)
**(b) Skin lesion (*n* = 11)**	2.91 ± 0.51 ^b^ (2.20–6.17)	8.05 ± 1.09 ^b^ (6.80–10.34)	244.4 ± 105.60 ^a^ (98.10–408.20)	587.35 ± 447.37 ^b^ (127–1444.40)	628.16 ± 412.69 ^b^ (64.80–1348.20)
**(c) Reference values range**	2.70–3.70	5.90–7.80	49–123.30	26.90–156.10	19.60–49.10

## Data Availability

Supporting data is available to applicants through the correspondence address.
